# Supplementary material for: Method comparison studies of telomere length measurement using qPCR approaches: A critical appraisal of the literature
Source: PLoS One. 2021 Jan 20;16(1):e0245582. doi: 10.1371/journal.pone.0245582 (PMC7817045; doi:10.1371/journal.pone.0245582)
Supplement: S1 Table — Reporting items of the TRN Guidelines by category, with assigned importance for each item and comparison with the related assigned importance of the MIQE guidelines. E: essential information, should be submitted with the manuscript. D: desired information, should be submitted if available. (DOCX) [file pone.0245582.s001.docx]

S1 Table. Telomere Research Network Reporting Guidelines.

| Category (score) | Reporting Item | Importance | MIQE Importance |
| --- | --- | --- | --- |
| Sample type, storage, DNA extraction and integrity (score out of 9 or 10) | Sample type | E | E |
|  | Sample storage temperature | E | E |
|  | Sample storage time before extraction | D | E |
|  | Sample storage buffer | D | E |
|  | DNA extraction method | E | E |
|  | DNA storage conditions | E | N/A |
|  | DNA freeze-thaw cycles | E | N/A |
|  | Method of documenting DNA quality/integrity | D | E |
|  | % of samples tested for DNA quality/integrity | D | N/A |
|  | **For studies with repeated measures design report the above for all time points* | E | N/A |
| qPCR assay (score out of 13 or 15) | State type of PCR method | E | N/A |
|  | PCR machine | D | E |
|  | Source of master mix & reagents | E | E |
|  | Final reaction volume | E | E |
|  | Telomere primer sequences | E | E |
|  | Telomere primer concentration | E | N/A |
|  | Single copy gene name | D | N/A |
|  | Single gene primer sequence | E | E |
|  | Singe gene primer concentration | E | N/A |
|  | Full PCR program description including temperature, times, and cycle numbers | E | E |
|  | PCR efficiency of single copy gene and telomere primers | E | E |
|  | Source of control samples | E | N/A |
|  | Concentration of DNA standard | E | N/A |
|  | *For aTL PCR measurement only: sequence of oligo standards* | E | E |
|  | *For aTL PCR measurement only: concentration of oligo standards* | E | N/A |
| Data analysis (score out of 12 or 13) | Mean and standard deviation or median and range of telomere lengths | D | N/A |
|  | Number of sample replicates | E | E |
|  | Level of independence of the replicates (plate vs day vs extraction) | E | E |
|  | Analytic method, considering replicate measurements, to determine final TL | E | N/A |
|  | Method of accounting for variation between sample replicates | E | N/A |
|  | Method of accounting for well position effects | E | N/A |
|  | Method of accounting for between-plate effects | E | N/A |
|  | % of samples repeated due to failed QC | D | E |
|  | % of samples excluded from analysis due to failed QC | E | E |
|  | Acceptable range of PCR efficiency for primers | D | N/A |
|  | ICCs of sample/study groups | E | N/A |
|  | T/S ratio transformed to Z score prior to analysis | D | N/A |
|  | *For studies with family samples or repeated measures design: analytic method to account for this* | E | N/A |
