# Supplementary material for: Method comparison studies of telomere length measurement using qPCR approaches: A critical appraisal of the literature
Source: PLoS One. 2021 Jan 20;16(1):e0245582. doi: 10.1371/journal.pone.0245582 (PMC7817045; doi:10.1371/journal.pone.0245582)
Supplement: S2 Table — Reporting items of the guidelines developed by Morinha et al. by category, with assigned importance for each item and comparison with the related assigned importance of the MIQE guidelines. A: very important. B: somewhat important. C: somewhat important, should be submitted if available. E: essential information, should be submitted with the manuscript. D: desired information, should be submitted if available. (DOCX) [file pone.0245582.s002.docx]

S2 Table. Morinha Reporting Guidelines.

| Category (score) | Reporting Item | Significance | MIQE Importance |
| --- | --- | --- | --- |
| Sample (score out of 5) | Experimental and control groups characteristics | A | E |
|  | Tissue sampled | A | E |
|  | Volume/mass of sample processed | B | D |
|  | Storage conditions in the field and lab (including freeze-thaw cycles) | A | E |
|  | Storage time before DNA extraction | B | E |
| DNA extraction (score out of 7) | Name of kit and details of any modifications | A | E |
|  | Procedure and/or instrumentation | A | E |
|  | Extraction method of the calibrator sample (if applicable) | A | N/A |
|  | DNA quantification method | B | E |
|  | DNA quality and purity (integrity, yield, 260/280 and 260/230 ratios) | A | D |
|  | Storage conditions | A | N/A |
|  | Freeze-thaw cycles before qPCR | B | N/A |
| qPCR validation (score out of 7) | Calibration curves with slope and y-intercept | A | E |
|  | R2 of calibration curve | A | E |
|  | qPCR efficiencies (telomere and reference genes) | A | E |
|  | Cq of the NTC | B | E |
|  | Linear dynamic range | B | E |
|  | Specificity (e.g. gel, sequence, melt) | A | E |
|  | Amplification and melting curve plots | B | N/A |
| qPCR protocol (score out of 12 or 13) | Primer sequences | A | E |
|  | PCR Master mix (manufacturer and name of the products used in the assay) | A | E |
|  | Complete reaction conditions (dNTP, MG2+, primer and polymerase concentrations, DNA amount, other components, reaction volume | A | E |
|  | Additives (SYBR Green I, ROX, DMSO, etc) | A | E |
|  | Calibrator sample used (e.g. synthetic, pool) | A | N/A |
|  | Singleplex or multiplex | A | N/A |
|  | *If singleplex, were both reactions run on the same plate or not* | A | N/A |
|  | Number of replicates (technical and biological) | A | E |
|  | Were different groups (age/sex/etc) run on the same plates or randomized | A | N/A |
|  | Manufacturer of plates/tubes and catalog number | C | D |
|  | Complete thermocycling parameters | A | E |
|  | Reaction setup (manual/robotic) | C | D |
|  | qPCR instrument | A | E |
| Data analysis (score out of 13) | Quality control steps for data | A | E |
|  | Analysis program (source, version) | A | E |
|  | Method used in the data analysis | A | E |
|  | Choice of reference genes | B | E |
|  | Concentrations of the calibrator sample used to create standard curves | A | N/A |
|  | Normalalization and adjustment methods | A | E |
|  | Repeatability (intra-assay variation) | A | E |
|  | Reproducibility (inter-assay variation statistics) | A | D |
|  | Acceptance and rejection criteria | A | E |
|  | How was telomere length calculated? | A | N/A |
|  | Statistical methods for results significance | A | E |
|  | Software used for statistical analysis (source, version) | A | E |
|  | Cq or raw data submission | B | D |
