# Supplementary material for: Method comparison studies of telomere length measurement using qPCR approaches: A critical appraisal of the literature
Source: PLoS One. 2021 Jan 20;16(1):e0245582. doi: 10.1371/journal.pone.0245582 (PMC7817045; doi:10.1371/journal.pone.0245582)
Supplement: S3 Table — (DOCX) [file pone.0245582.s003.docx]

S3 Table. Reporting guidelines rubric.

| **For all reporting guidelines:** | - Record information as reported only if it is provided directly in the paper itself or in the 1^st^ order of references cited (with exceptions related to Cawthon papers – see below). Do not include information that extends beyond one previous citation. For example, if the methods section states that the detailed methods are described in a previous paper, report only the information from that cited paper. If the referenced paper itself has an additional reference for methods, mark this information as not provided.   - Applies for both methods and sample demographics (i.e. if further analysis is done on samples from a previously published study) - Methods sections that state “followed Cawthon 2002 and/or 2009” and failing to provide any additional information about the specific assay performed are not counted as reporting assay parameters and conditions - If sample demographics for the overall population in the study are provided, and only a subset of samples were involved in cross method analyses, reviewer can consider the demographic characteristics as present for the cross-method sample - Information in supplemental material or supplemental methods is reported as present. - qPCR efficiencies in qPCR assay/validation section: this refers to actual efficiencies of the assays performed |
| --- | --- |
| **TRN reporting guidelines:** | - Studies without repeated measures design: do not include “for studies with repeated measures….” in score for sample type, storage, extraction, and integrity (mark N/A) or “method of accounting for within-family samples or repeated measures design” if it does not apply (mark N/A, exclude from score) - Studies without aTL measurement: mark N/A for “for aTL PCR measurement…” and do not include in score - DNA quality and integrity: report as yes if methods section describe how DNA quality was assessed; actual DNA values not required (note this differs from the Morinha requirement) - Acceptable range of PCR efficiency: Note this differs from actual PCR efficiency values in the experiment, and should express the lab’s typical acceptability/exclusion criteria for PCR assay efficiency |
| **Morinha reporting guidelines:** | - Experimental and control group characteristics: if cross-method analysis is performed on only a subset of samples, report this as present only if characteristics are provided specifically for the subset of samples (not just the entire experiment) - DNA quality and purity: report yes only if actual DNA integrity, yield, 260/280, 260/230 ratios are provided (ranges/mean are acceptable) - Choice of reference genes: Name of single-copy gene |
