# Supplementary material for: Method comparison studies of telomere length measurement using qPCR approaches: A critical appraisal of the literature
Source: PLoS One. 2021 Jan 20;16(1):e0245582. doi: 10.1371/journal.pone.0245582 (PMC7817045; doi:10.1371/journal.pone.0245582)
Supplement: S4 Table — Scores for each category are provided as a percentage of items in that category sufficiently reported. A total score for each set of reporting guidelines are calculated from all items as well as an average overall score between the two reporting guidelines. (DOCX) [file pone.0245582.s004.docx]

**S4 Table. Results of reporting guidelines for individual papers.**

| Author | TRN Reporting Guidelines | | | | Morinha Reporting Guidelines | | | | | | ***Average Overall Grade*** |
| --- | --- | --- | --- | --- | --- | --- | --- | --- | --- | --- | --- |
|  | Sample | qPCR assay | Data analysis | ***Overall Grade*** | Sample | DNA extraction | qPCR validation | qPCR protocol | Data analysis | ***Overall grade*** |  |
| Hsieh | 44% | 92% | 58% | 68% | 40% | 71% | 71% | 85% | 92% | 78% | **73%** |
| Eisenberg | 67% | 85% | 58% | 71% | 40% | 86% | 29% | 83% | 85% | 70% | **71%** |
| Zanet | 56% | 92% | 50% | 68% | 80% | 29% | 43% | 77% | 92% | 69% | **68%** |
| Martin-Ruiz (b) | 50% | 92% | 77% | 75% | 40% | 57% | 0% | 62% | 100% | 60% | **68%** |
| Tarik | 56% | 92% | 58% | 71% | 40% | 29% | 71% | 75% | 77% | 64% | **67%** |
| Jodcyzk | 67% | 92% | 58% | 74% | 40% | 43% | 0% | 69% | 77% | 53% | **63%** |
| Gutierrez-Rodrigues | 78% | 77% | 42% | 65% | 60% | 57% | 0% | 77% | 77% | 60% | **62%** |
| Ropio | 67% | 67% | 33% | 56% | 80% | 71% | 0% | 69% | 85% | 64% | **60%** |
| Imam | 33% | 77% | 33% | 50% | 80% | 29% | 14% | 77% | 69% | 58% | **54%** |
| Ehrlenbach | 30% | 46% | 69% | 53% | 20% | 43% | 0% | 62% | 83% | 49% | **51%** |
| Aviv | 33% | 85% | 25% | 50% | 40% | 14% | 0% | 69% | 69% | 47% | **48%** |
| Gadalla | 56% | 69% | 25% | 50% | 60% | 29% | 0% | 54% | 54% | 42% | **46%** |
| Khincha | 22% | 69% | 42% | 47% | 40% | 14% | 0% | 54% | 69% | 42% | **45%** |
| Wang | 22% | 69% | 42% | 47% | 40% | 14% | 0% | 46% | 54% | 36% | **41%** |
| Panero | 11% | 73% | 18% | 40% | 40% | 0% | 43% | 77% | 31% | 42% | **41%** |
| Behrens | 33% | 62% | 33% | 44% | 40% | 29% | 0% | 38% | 62% | 38% | **41%** |
| Lee | 11% | 69% | 17% | 35% | 40% | 0% | 0% | 69% | 38% | 36% | **35%** |
| Pavesi | 11% | 77% | 8% | 35% | 40% | 0% | 0% | 54% | 46% | 33% | **34%** |
| Wand | 11% | 46% | 25% | 29% | 40% | 29% | 0% | 38% | 54% | 36% | **32%** |
| Ventura Ferreira | 22% | 31% | 25% | 26% | 40% | 29% | 0% | 25% | 46% | 29% | **28%** |
| Gardner | 44% | 92% | 25% | 56% | 40% | 43% | 0% | 77% | 77% | 56% | **56%** |
| Hunt | 11% | 92% | 33% | 50% | 40% | 0% | 0% | 85% | 85% | 53% | **52%** |
| Martin-Ruiz (a) | 22% | 85% | 33% | 50% | 20% | 33% | 0% | 69% | 31% | 36% | **43%** |
| Salpea | 22% | 92% | 50% | 56% | 40% | 29% | 71% | 69% | 85% | 60% | **58%** |
| Lynch | 33% | 46% | 42% | 41% | 80% | 29% | 0% | 78% | 69% | 49% | **45%** |
| **Average per Category** | **37%** | **75%** | **39%** | **52%** | **46%** | **32%** | **14%** | **66%** | **68%** | **50%** | **51%** |
