# Supplementary material for: Method comparison studies of telomere length measurement using qPCR approaches: A critical appraisal of the literature
Source: PLoS One. 2021 Jan 20;16(1):e0245582. doi: 10.1371/journal.pone.0245582 (PMC7817045; doi:10.1371/journal.pone.0245582)
Supplement: S1 File — (DOCX) [file pone.0245582.s006.docx]

**Supplemental Methods**. Calculation of the repeatability (ICC) of telomere length measures.

Unlike the CV statistics, the ICC cannot be calculated for each biological sample individually, but instead is calculated for a set of biological samples. Thus, to be able to calculate the repeatability, (a subset of) samples should be re-measured one or more times in an identical fashion as TL is typically measured in one’s laboratory. Two important considerations when selecting these samples are the following. Firstly, variation in TL measurement can arise at any point between sample collection and arrival at your TL estimate. Therefore, the ICC will be overestimated when, for example, the ICC is estimated over repeated measurements of extracted DNA relative to measurements where the DNA extraction is also repeated (but by how much is not known). Thus, the ICC you estimate from the data will be closer to the true ICC when more of the measurement process is repeated independently in the successive measurements. Secondly, the TL of the subset of samples needs to be representative of the complete set of samples that is analysed. This is true in particular for the range of TL in the sample – having a larger range in the subset than in the ultimate sample will overestimate the ‘true’ ICC, while having a smaller range in the subset than in the ultimate sample will result in underestimation of the ICC.

The ICC can be calculated in different ways, depending on how variation between batches is accounted for (i.e. not at all, as random effect, or as fixed effect), where ‘batches’ can be thought of as different plates, or gels, or measurement sessions that differ in any respect (e.g. day, person, equipment, laboratory). The best way to account for batch in the ICC calculation is to use the same approach as in the ultimate analyses in which hypotheses are tested.

The text below is an R script, mixing instructions with the actual script. When a line is precede by '#', this indicates it is a comment – and it will not be executed. It is left in the text here to make it possible to copy all text below (up to the References) to an R-script – see instructions below.

# R script to calculate the ICC (IntraClass Correlation), also known as 'repeatability'.

# The text below assumes you are new to R....

# '#' before text indicates it is a comment - will not be executed

# The other lines need to be 'run' and results will show in the console window.

# Before you start

# 1. it is advisable to do the analysis in Rstudio (freely downloadable and works on

# multiple platforms), which serves as a 'shell' to R, and copy this text to a new R-script.

# 2. A useful introduction by the authors of the package used in this script to calculate

# the ICC is recommended reading:

# https://cran.r-project.org/web/packages/rptR/vignettes/rptR.html

# The text below is only enough to get you started

# 3. Data format

# The data need to be in the 'long' format. This implies ALL the telomere estimates are

# in one column, with sample identity in another column. Data may have been collected in

# different 'batches' (plates, gels, days, labs) and batch identity is coded in a separate

# column.

# When your data is in wide format, this can easily be changed to a long format in Excel.

# So the data file usually has a minimum of three columns (with variable names in brackets):

# sample identity (id), telomere estimate (TL), batch (batch) [when you use different

# variable names, the names in the code below need to be replaced with those names].

# The packages listed below are needed and you will probably need to install them first.

# At the top of the bottom right panel in RStudio there is a tab 'packages' you can use.

library(readxl) #for when your data are in Excel format

library(MASS)

library(lme4)

library(rptR)

# To clear lists of objects – useful to run whenever you start an analysis:

rm(list=ls())

rm(list = ls(all = TRUE))

# Reading in the data.

# In the example below, the data were stored in Excel, but Rstudio reads many formats.

# Other formats may require loading another package - Rstudio will tell you this.

# Note that the first bit of the code below, 'd <-', you can read as 'd becomes'.

# We here arbitrarily name the data set 'd' (commands / names in R are case sensitive!).

# You can get the import code for your file location and correct command for your file format

# using the 'import dataset' tab in RStudio (above the top right frame on a mac).

# When you import a data set using RStudio, the dataset will have a name different from

# what is in the script below. I recommend copying the code you see after the "<-" to the

# script just before actually importing the data and copy this below after the text "d <-".

# Alternatively, you replace 'd' in the code with the name you have given your data set.

d <- read_excel("FILE LOCATION/example.xls") #importing the example data from Excel file

# The file 'example.xls' is available on the telomere network site.

# Calculating the ICC

# The ICC can be calculated in different ways, depending on how variation between

# batches is accounted for (i.e. not at all, as random effect, or as fixed effect).

# The best way to account for batch in the ICC calculation is to use the same approach

# as in the analysis for which the data were collected.

# Below is the code for different ways to include 'batch' in the analysis.

# When running the scripts below, depending on details,

# there may be 'Singularity' issues that are reported as errors.

# You can safely ignore these (see information on rptR package for details.)

# 1. No correction for batch

# Including 'id' only - i.e. batch is not in the model

# Note that the (1|something) codes for a random intercept for levels of 'something'

rpt(TL ~ (1|id), grname = "id", data=d, datatype = "Gaussian", nboot = 1000, npermut=0)

# 2. Including 'batch' as random effect

rpt(TL ~ (1|id) + (1|batch), grname = "id", data=d, datatype = "Gaussian", nboot = 1000, npermut=0)

# 3. Including 'batch' as fixed effect

rpt(TL ~ batch + (1|id), grname = "id", data=d, datatype = "Gaussian", nboot = 1000, npermut=0)

# Note that these models can be extended with other factors and covariates.

# See example below that includes age (as fixed effect)

rpt(TL ~ age + (1|id) + (1|batch), grname = "id", data=d, datatype = "Gaussian", nboot = 1000, npermut=0)

# When running the model including age, the ICC is likely to become lower. The new (lower)

# ICC estimate is the more relevant estimate. This is so, because you will probably

# take age into account in your statistical analyses also. The remaining variation in the

# data will decrease as a consequence, and the ICC is calculated over the remaining variation.

# Adding other factors to the model, e.g. batch identity or procedure characteristics

# that you also include in the model with which you test hypotheses can also be added as

# factors to the model, and may increase the ICC.

# The script above assumes the TL measurements are normally distributed, but the

# rptR package can handle other distributions.

# Extrapolated repeatability – an important extension!

# Suppose your protocol includes rTL measurement in duplicate, using for example two plates

# with samples in triplicate on each plate, and you use the average of the two plates in

# the data analysis. You can then calculate the ICC over the two plates, but this will

# underestimate the ICC of the average of the two plates, which is based on more measurements.

# However, the ICC of the mean of the two plates can be calculated with a simple equation

# once the ICC over the two plates is known: when r is your repeatability (ICC) estimate,

# and n is the number of replicate measurements then the extrapolated repeatability (re):

# re = r / (r+1/n*(1-r))

# This is equation 37 in: Nakagawa, S. & Schielzeth 2010, Biological Reviews 85, 935-956.
